# The Role of IL-6 and ET-1 in the Diagnosis of Coronary MicroVascular Disease in Women

**DOI:** 10.3390/jpm11100965

**Published:** 2021-09-27

**Authors:** Diana Gurzău, Adela Sitar-Tăut, Bogdan Caloian, Gabriel Guşetu, Horaţiu Comşa, Florina Frîngu, Dumitru Zdrenghea, Dana Pop

**Affiliations:** Cardiology Department, Clinical Rehabilitation Hospital, “Iuliu Hațieganu” University of Medicine and Pharmacy, 400012 Cluj-Napoca, Romania; gurzaudiana@yahoo.com (D.G.); adelasitar@yahoo.com (A.S.-T.); gusetu@gmail.com (G.G.); dh.comsa@gmail.com (H.C.); florina.fringu@yahoo.com (F.F.); dzdrenghea@yahoo.com (D.Z.); pop67dana@gmail.com (D.P.)

**Keywords:** ischemic heart disease in women, microvascular angina, IL6, ET1

## Abstract

Background: Microvascular angina is a common clinical entity, with about a three-fold higher frequency in women. The pathogenesis of microvascular angina has not been much studied, but inflammation and endothelial dysfunction have been incriminated as the main mechanisms of this disease. Methoss: Our purpose was to analyze whether certain inflammatory markers, i.e., interleukin 6 (IL-6) and endothelin 1 (ET-1), can play a role in the diagnosis of microvascular angina in women. Results: Ninety women with ischemic heart disease were divided into two groups, based on their affliction with either microvascular or macrovascular disease. In general, the levels of IL6 and ET1 were similar between the two groups. Analyzing these marker levels according to the number of coronary lesions, we obtained an increased IL6 value that was similar for patients with microvascular angina, one-vessel, and two-vessel coronary disease, but significantly lower than in women with three-vessel coronary lesions. Also, in microvascular angina, IL6 level was correlated with the NYHA IV functional class. Unexpectedly, the level of ET1 was correlated with left ventricular systolic dysfunction. Conclusions: In women with an increased suspicion of microvascular angina, in whom microvascular dysfunction cannot be tested invasively, IL-6 level, unlike the ET-1 level, might be considered a diagnostic marker of this disease.

## 1. Introduction

Ischemic heart disease through microvascular involvement is a common clinical entity, with about a three-fold higher frequency in as women compared with men [1]. The pathogenesis of coronary microvascular disease has not been much studied, as it is a somewhat newer clinical entity. Coronary microvascular involvement refers to coronary microcirculation that is not visible angiographically and comprises all vessels (prearterioles, arterioles, capillaries) with a diameter smaller than 0.4–0.5 mm, circulation that represents about 95% of coronary vascularization and is, in fact, the main site of blood flow self-regulation as well as vascular resistance [2]. These self-regulation mechanisms ensure myocardial blood flow, including in situations of obstruction or significant stenosis of the epicardial arteries, through the compensatory vasodilation of microvascular circulation [2]. When this compensatory vasodilation process is inefficient or overcome, it results in a reduction of coronary flow reserve, with the development of ischemia.

The causes considered responsible for deficient vasodilation would be anatomical microcirculation abnormalities, vascular spasm at this level, or the presence of endothelial dysfunction [3,4]. From a practical point of view, the diagnosis of microvascular angina is most frequently established by coronarography, where following visualization of normal epicardial arteries, the coronary microcirculation physiology is assessed by determining coronary flow, flow reserve, as well as vascular resistance. Invasive testing of microvascular dysfunction is a procedure that is not routinely performed. This procedure is technically extremely laborious, requiring special software and, of course, an experienced doctor; it is also time consuming, increasing the duration of the procedure by up to 30 min and is much more expensive. Due to this fact, we need other clinical and paraclinical parameters to help us establish the diagnosis of microvascular coronary dysfunction [5]. 

The presence of endothelial dysfunction plays a primary role in the development of this particular form of ischemic heart disease. This involves an imbalance of vasodilator and vasoconstrictor peptides, in favor of peptides with a vasoconstrictive effect, of which ET-1(endothelin-1) is the most important. ET-1 is the most powerful endogenous vasoconstrictor produced by vascular endothelium, involved in the occurrence of endothelial dysfunction and inflammation, but also in the process of vascular remodeling [6,7]. ET-1 acts through receptors that are bound to two G proteins, namely endothelin A-ETA receptor and endothelin B-ETB receptor. The effects of ET-1 coupling with the two receptors differ, so by binding to ETA receptors, located mainly in smooth muscle vascular cells, vascular vasoconstriction is mediated, and coupling to ETB receptors, located mainly in endothelial cells, mediates vascular vasodilation [7,8] However, the coupling with the ETA receptor has a greater clinical importance, importance as this coupling is involved in the occurrence of hypertension, atherosclerotic disease, consequently cardiovascular diseases; more, it seems that it has a role in the pathogenesis of chronic kidney disease and in diabetes, especially its vascular complications [7,8]. Its role in the diagnosis and prognosis of patients with microvascular angina has been very little studied. Most of the studies evaluating and confirming the role of endothelin as a mediator of microvascular dysfunction have focused on microvascular dysfunction secondary to percutaneous revascularization in patients with acute coronary syndromes [9], but there are extremely few studies that assess this role in patients with microvascular angina alone. 

Alongside endothelial dysfunction, inflammation also plays an extremely important role in coronary microcirculation, with pertinent consequences (there is a close interaction between the two processes) [6]. Inflammation, through the presence of cytokines such as IL-6 (interleukin 6), C reactive protein, can also underlie coronary microvascular endothelial dysfunction in patients with microvascular angina. IL-6 is a cytokine derived from T lymphocytes, macrophages, and fat cells, having a major role in triggering and maintaining the inflammatory cascade by stimulating fibrinogenesis and stimulating C-reactive protein, with consequences for initiating and accelerating the atherosclerotic process [10,11]. Interleukin practically stimulates the hepatic production of acute phase proteins, such as those mentioned above, proteins that are known to be strongly involved in the pathogenesis of ischemic coronary heart disease. Also, another link in the pathogenetic chain induced by IL6 that underlies the occurrence of ischemic heart disease is represented by the increase of blood viscosity, with the activation and proliferation of platelets. Subsequently, at the level of the vascular wall, by activating the monocytes by IL6, the fibrinogen is deposited, after which by inhibition also induced by IL6, the activity and the plasma level of lipase lipoproteins decrease, resulting in the increase of macrophage lipid absorption. Finally, interleukin 6 will activate the hypothalamic-pituitary-adrenal axis, which will contribute to the onset and maintenance of atherosclerosis, hypertension, obesity, and insulin resistance [11,12]. So far, there are no studies analyzing the role of interleukin 6, consequently inflammation, in the diagnosis and prognosis of patients with ischemic heart disease through microvascular involvement, and even less so in female patients. 

In this context, the current study aims to analyze whether certain inflammatory markers, i.e., interleukin 6 (IL-6) and endothelin 1 (ET-1), can play a role in the diagnosis of ischemic heart disease through microvascular involvement in women.

## 2. Materials and Methods

The study included 90 patients diagnosed with ischemic heart disease, admitted to the Clinical Rehabilitation Hospital Cluj-Napoca, Romania, with a mean age of 69.02 ± 9.34 years.

Ischemic heart disease is defined according to the 2019 recommendations of the diagnostic and treatment guidelines for chronic coronary syndromes of the European Society of Cardiology, based on clinical symptoms, rest ECG, exercise testing, echocardiography and coronarography [13]. Thus, for coronary macrovascular disease, coronary lesions were considered significant for stenosis > 70% or >50% in the case of the main trunk [13]. The diagnosis of microvascular angina was made based on typical angina symptoms, correlated with electrocardiographic rest changes, positive exercise test, and coronarographically normal epicardial arteries with delayed coronary flow of the contrast substance. We mention that the invasive testing of microvascular dysfunction, by evaluating vasoreactivity, coronary blood flow and coronary flow reserve is not performed routinely, being a very expensive and time-consuming procedure. Precisely for these reasons, we wanted to evaluate other clinical and paraclinical parameters to help us diagnose coronary heart disease.

Cardiovascular risk factors and echocardiographic parameters were evaluated for all patients. Also, all women underwent coronarography. The patients included in the study were divided into two groups: the first group with coronary microvascular disease (50%) or microvascular angina, and the second group was represented by patients with macrovascular atherosclerotic coronary lesions (50%). 

The venous blood samples required for the measurement of serum IL-6 and ET-1 concentrations were taken on the day of admission, after obtaining the patients’ informed consent regarding biological sample collection for research purposes and the confidentiality of the results obtained. By centrifugation at 1500× *g*, at a temperature of 4 °C for 10 min, the serum was separated from the rest of the blood composition, and subsequently this was transferred to 1 mL cryotubes and stored at a temperature of −70 °C. Both IL-6 and ET-1 concentrations were measured using the ELISA method. Test calibration was performed according to the manufacturer’s recommendations. The reference interval for ET-1 was between (1–3) pmol/L, and a value considered above the normal limit of IL-6 was ≥6.5 pg/mL. For CRP (C-reactive protein), normal value was considered to be less than 0.6 mg/dL. Regarding the plasma concentration of IL-6, a single extreme value of 500 pg/mL was recorded, this being found in a patient with a very important atherosclerotic load, respectively the patient present with triple vessel coronary heart disease (LAD, CX, RCA), with percutaneous revascularization but with repeated stent thrombosis, with endarterectomy on the right internal carotid artery, with repetitive ischemic stroke, grade III arterial hypertension, major valvulopathies, congestive heart failure, obesity and gastroesophageal reflux disease.

Echocardiographically, the telediastolic, telesystolic diameters and the left atrial diameter in the parasternal long axis view were measured, the LV ejection fraction (LVEF%) was calculated using the biplane Simpson’s method, which was considered normal at a value higher than 50%, diastolic dysfunction was assessed, and the presence of parietal kinetic disorders was also recorded. 

The exclusion criteria were represented by the presence of acute or chronic infections, systemic or autoimmune diseases, ongoing or previous neoplastic pathology and recent surgical procedures 

Statistical analysis was performed in Writer, Presentation and Spreadsheets (WPS) Office 2019. For all statistical analyses and subsequent diagrams, R 4.0.0 was used. We considered that *p* < 0.05 was statistically significant and *p* < 0.10 only showed a tendency to statistical significance.

The selected patients were informed about the study protocol and signed an informed consent form. The present clinical study was approved by the local ethics committee (approval number 2606/4.04.2018) and was performed in accordance with the ethical standards established by the 1964 Declaration of Helsinki and its later amendments.

## 3. Results

The general characteristics of the patients included in the study are represented in Table 1. As the table shows, there were no significant differences between the two groups regarding age, associated risk factors, symptoms, except for palpitations which were frequently found in women with coronary microvascular disease. In fact, atrial fibrillation was present in a significantly higher proportion in these women compared to those with macrovascular involvement (53.3 vs. 66.66%, *p* = 0.0201). On the other hand, women with coronary macrovascular disease more frequently had a history of acute coronary syndromes and heart failure.

When analyzing the correlation between the serum levels of inflammatory biomarkers, depending on the degree of coronary involvement, expressed by the number of coronary lesions, we observed a statistically significant difference in the group of patients with multivascular coronary lesions (Table 2). The IL-6 value was significantly higher in this group compared to patients with one-vessel or two-vessel coronary involvement, as well as to patients with microvascular angina. The IL-6 value was similar in the group of patients with microvascular angina and in the group of patients with macrovascular involvement, with one-vessel and two-vessel coronary lesions.

There were no differences in ET-1 levels between the two groups or among patients with macrovascular involvement, regardless of the number of affected vessels.

**Figure 1:**
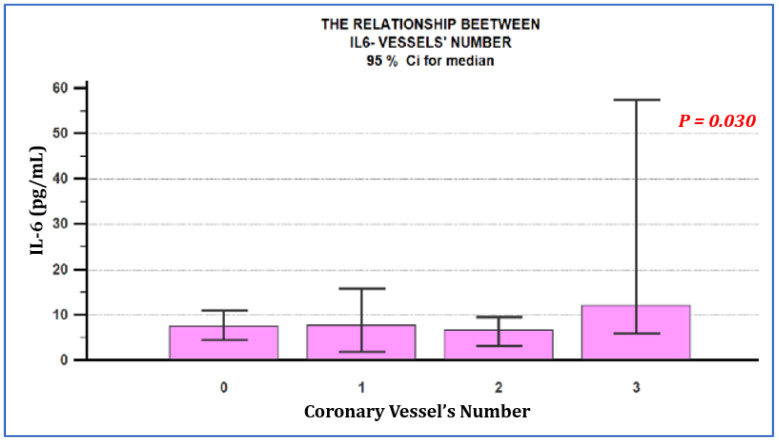
Relationship between the serum IL-6 value and the number of affected coronary vessels. IL-6 levels were similar in the group of patients with microvascular angina, one and two-vessel coronary artery disease, but statistically significantly higher among patients with three-vessel coronary lesions (*p* = 0.030). 0—microvascular angina; 1—one-vessel coronary disease; 2—two-vessel coronary disease; 3—three-vessel coronary disease.

**Figure 2:**
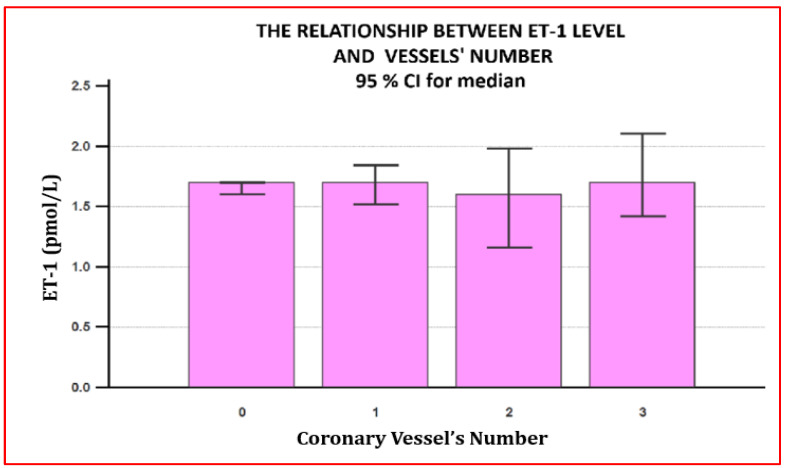
The relationship between plasma ET-1 concentration and the number of coronary lesions. The plasma concentration of ET-1 did not show significant differences either in terms of the type of coronary heart disease, microvascular versus macrovascular, either in terms of the number of coronary arteries involved for patients with obstructive coronary heart disease. 0—microvascular angina; 1—one-vessel coronary disease; 2—two-vessel coronary disease; 3—three-vessel coronary disease.

These details are presented in Table 2, Figure 1 and Figure 2.

IL-6 and ET-1 values were not correlated with the LDL cholesterol level recorded in the two groups of patients, for patients in the group with microvascular involvement Rho −0.185, *p* = 0.283 versus Rho −0.122, *p* = 0.4181 for patients with macrovascular involvement. 

When analyzing the relationship between IL-6 level and age, a statistical correlation was observed between these two variables, but when evaluating this interrelation comparatively between the two groups of patients, the IL-6 value was correlated with age only in the first group, of patients with microvascular involvement (Rho 0.301, *p* = 0.0459), not in the second group (Rho 0.230, *p* = 0.1277). Regarding the ET-1 value, this was not correlated with patients’ age in the general population of the study or with coronary involvement. Table 3 shows data regarding the correlation between the values of biomarkers and patients’ age.

**Figure 3:**
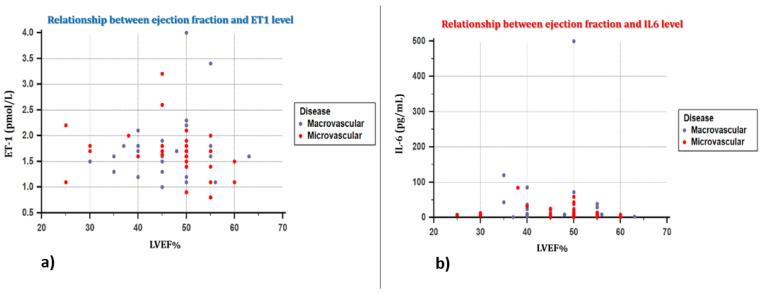
(**a**) Correlation between the ET-1 level and the ejection fraction value in the two groups. ET-1 levels were statistically significantly correlated with LVEF% values in patients with microvascular disease, but not in patients with obstructive coronary heart disease. (**b**) Graphic representation of the correlation between the IL-6 value and the ejection fraction value. Serum IL-6 concentration was not correlated with LVEF%, regardless of the type of coronary heart disease. LVEF%-left ventricular ejection fraction.

By analyzing the influence of IL-6 and ET-1 markers on systolic ventricular function, assessed through the left ventricular ejection fraction value, and on diastolic function, we obtained statistically significant correlations only in the group of patients with microvascular angina, where the ET-1 level was statistically significantly associated with the left ventricular ejection fraction value (Rho −0.440, *p* = 0.0035). All statistical data are presented in Table 4, Figure 3a,b.

In contrast, inflammation assessed based on the IL-6 and ET-1 value was not correlated with the presence of atrial fibrillation in the group of patients with microvascular involvement or in the group of patients with macrovascular coronary lesions (Table 5).

Table 6 and Table 7 illustrates the correlations between the studied biomarkers and the presence of associated comorbidities depending on the type of coronary involvement.

**Figure 4:**
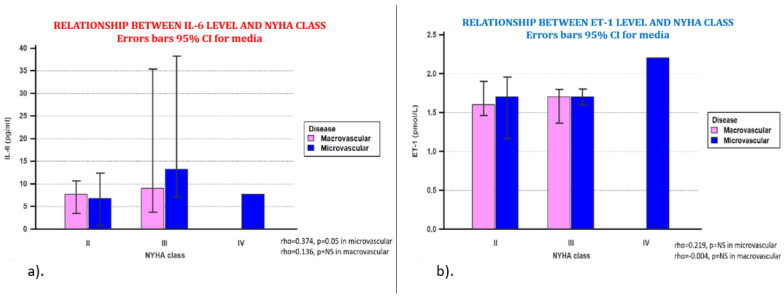
(**a**). Relationship between IL-6 value and NYHA functional class comparatively between the two groups. The increased value of IL6 was directly associated with NYHA functional class, but only for patients with microvascular angina, not for those with coronary heart disease. (**b**). Relationship between ET-1 level and NYHA functional class comparatively between the two groups. The ET1 value was not correlated with the NYHA functional class, regardless of the type of coronary heart disease, microvascular or macrovascular. NYHA class—New York Heart Association, Functional Classification.

There was a statistically significant correlation between the IL-6 level and the NYHA functional class only in the group of patients with microvascular involvement (Figure 4a). Regarding the relationship between the ET-1 value and the NYHA functional class, no statistical correlation was found regardless of the type of coronary involvement (Figure 4b).

## 4. Discussion

It is well known that coronary disease with normal epicardial arteries has been diagnosed in an extremely low proportion in specialized studies. Thus, in the CRUSADE registry, only 8.6% of patients had myocardial infarction without ST elevation, with normal coronary arteries [14]. Also, myocardial infarction with normal epicardial arteries is found in a much higher proportion in women than in men. The latest findings presented in the GUSTO IIb trial show that myocardial infarction without obstructive coronary lesions was detected in 22% of women versus only 10% of men, which can mainly be explained by the presence of endothelial dysfunction [15]. In the current study, we attempted to evaluate the role of some markers of inflammation (IL-6 and ET-1) in women diagnosed with microvascular angina compared to those diagnosed with macrovascular angina.

Thus, we found in the first place that between the two forms of coronary involvement, there were no differences regarding the values of lipid fractions, uric acid, CRP, IL-6, or ET-1. This result is extremely important, practically demonstrating the not-so-benign nature of ischemic heart disease through microvascular involvement, as it was believed, until recently, to be. Dyslipidemia plays a central role in the pathogenesis of atherosclerotic disease, the control of dyslipidemia being one of the main pillars of the therapeutic strategy in ischemic coronary macrovascular disease. However, when speaking about the role of dyslipidemia in the pathogenesis of microvascular angina, things are not so clear. So far, there are no studies describing the direct implication of dyslipidemia in the pathophysiology of microvascular angina. An indirect role can be attributed to dyslipidemia through secondary inflammation, which is known to play an important role in the development of microvascular angina. This aspect also explains the importance of treatment with statins in patients with microvascular angina [16]. The relationship between uric acid and ischemic coronary macrovascular disease is relatively well known, hyperuricemia increasing the risk of cardiovascular diseases through endothelial dysfunction secondary to oxidative stress, with a reduction of the nitric oxide level, blood platelet activation and an increase in the release of circulating cytokines, these processes underlying the initiation and perpetuation of the atherosclerotic process [17]. At the same time, women with ischemic coronary disease with associated hyperuricemia have adverse cardiovascular events and higher mortality rates [18]. Also, hyperuricemia, per se, represents an inflammatory state, so that uric acid may further contribute to the development of microvascular angina. 

Literature studies have confirmed the interrelation between high IL-6 levels and coronary disease, as well as the role of IL-6 assessment for the early detection of patients with extensive coronary lesions. It has also been demonstrated that, including at values > 1 pg/mL, in patients with intermediate cardiovascular risk, IL-6 can be an important predictor for ischemic coronary disease [19]. All these aspects are more studied in relation to coronary macrovascular disease compared to the implications of IL-6 level in the pathogenesis of coronary microvascular disease. So far, there are extremely few studies analyzing the direct implications of IL-6 in the development of microvascular angina, but it seems that in women with microvascular angina, there are eight inflammatory biomarkers that are associated with microvascular angina, and the majority of biomarkers have followed the pro-inflammatory CRP, TNF-α—IL-6 pathway [20]. Consequently, the results of our study confirm the previously mentioned statements. Women in the first group, similarly to those of the group with macrovascular involvement, had increased IL-6 values, much above the normal limit. Given the high similarity of the values between the two groups, it could be said that in microvascular angina, endothelial dysfunction expressed by the ET-1 level is as important as in atherosclerotic coronary disease. There are even studies that have identified a mutation/dysregulation of ET-1 in patients with microvascular angina and support the possibility of using gene therapy with the site of action at the level of the ETA receptor in these patients.

At the same time, although the plasma concentration of CRP was increased in both groups, the mean values were similar, with no statistically significant difference. This result confirms that inflammation is equally present in both forms of coronary heart disease. Much is known about the relationship between CRP and obstructive coronary heart disease, CRP is not only an excellent biomarker for detecting inflammation, but is also directly involved in the pathogenesis of atherosclerosis. Elevated CRP levels are associated with an increased risk of myocardial infarction, peripheral artery disease, sudden cardiac death and ischemic stroke, and, compared to other inflammatory biomarkers, it does not show diurnal variations, remaining stable for long periods of time. More importantly, the increased plasma concentration of CRP demonstrated a specificity in predicting the risk of cardiovascular disease [21]. 

However, regarding the implications of CRP in microvascular dysfunction, although the information in the literature is not so numerous compared to obstructive coronary disease, it seems that CRP is an important predictor of microvascular dysfunction, with inflammation playing a central role in the pathophysiology and complications of microvascular heart disease [22]. At the same time, reducing the plasma concentration of CRP by treatment with statins and/or antiplatelet agents seems to improve microvascular dysfunction [23].

By analyzing the correlation of IL-6 with the degree of coronary involvement, we observed that the IL-6 level, therefore a more significant inflammatory process, was found only in patients with multivascular atherosclerotic lesions. In contrast, the IL-6 level was similar in the group of patients with microvascular angina, with one-vessel and two-vessel coronary lesions. This aspect suggests, on the one hand, marked inflammation in microvascular angina, comparable to one-vessel and two-vessel atherosclerotic coronary disease, and so a less benign nature of microvascular angina. This is the first study that compares the degree of inflammation represented by the IL-6 level between the two types of ischemic coronary disease. On the other hand, the results suggest that a significantly increased IL-6 level is a predictor of multivascular coronary disease, which confirms the literature data, namely that a high IL-6 level is associated with a poorer prognosis, a higher risk of infarction and higher cardiovascular mortality [20].

However, ET-1 levels were not associated with the type of ischemic heart disease or with the degree of atherosclerotic coronary involvement. This result is somewhat surprising, in the first place because we did not expect the mean ET-1 value to be similar between the two groups but, rather, to be higher in the group of patients with atherosclerotic disease, and, in the second place, we expected ET-1 in the second group to be directly proportional to the number of coronary lesions given the direct implication of ET-1 in the atherosclerotic process. The results obtained in our study contradict the literature data, which demonstrate that the ET-1 level is associated with an advanced atherosclerotic process and progressive ischemic coronary disease [24]. Also, the ET-1 level is a predictor of mortality in patients with trivascular coronary disease [24]. Although we have no explanation for the absence of the differences in plasma ET-1 levels between the two groups, we can conclude that endothelial dysfunction among patients with microvascular angina is as expressed as in the case of coronary macrovascular disease. Consequently, these patients show, in time, an increased risk of developing an atherosclerotic process and macrovascular coronary lesions.

It is well known that, with aging, the risk of cardiovascular diseases increases. In women with menopause the pro-inflammatory status becomes more pronounced and the risk of ischemic heart disease increasing significantly. However, when analyzing the correlation of inflammatory markers with age, the correlation was found only among patients in the first group, with microvascular angina. Therefore, in women with microvascular angina, the slightly higher IL-6 level obtained was also due to the direct correlation with age and with the consequences of aging. In contrast, in the presence of coronary macrovascular disease, inflammation expressed by the IL-6 level is not influenced by age, being independent of it. Regarding the relationship between the plasma ET-1 level and age, studies demonstrate a direct causality between this and the development of cardiovascular diseases [25]. However, it is not yet clear whether the increase in ET-1 concentration is secondary to pathologies that develop with aging or if ET-1 concentration secondary to aging is causal for disease progression. It is certain that in addition to being involved in the aging process and in chronic diseases, ET-1 plays an extremely important role in the physiological processes of the organism [26]. In our study, the ET-1 level was not correlated with patients’ age; consequently, the plasma ET-1 concentration was most probably influenced by the associated ischemic pathology.

We found in our study that patients with microvascular angina had atrial fibrillation in a statistically significantly higher proportion. So far, there are no studies that compare the incidence of atrial fibrillation depending on ischemic coronary disease, microvascular and macrovascular, as it is well known that both forms are important etiologies of atrial fibrillation. Neither the IL-6 nor the ET-1 levels were correlated with the presence of atrial fibrillation in either of the two groups. This result could be explained by the small number of cases with associated atrial fibrillation, more precisely 53.3%, as well as by the lower level of the biomarkers, particularly ET-1, while IL-6 has proved to be an important mediator in the pathophysiological process of atrial fibrillation [27]. In its turn, the ET-1 level increases in patients with myocardial ischemia, which results in myocyte hypertrophy, myocardial fibrosis, and atrial dilatation. All this leads to an increase in atrial wall stress and to an alteration of atrial geometry, with consequences for electrical stability and the development of atrial fibrillation. Thus, the increased plasma ET-1 level is a strong and independent predictor of atrial fibrillation, predominantly among patients with ischemic coronary disease and heart failure [28]. 

An analysis of other comorbidities, in our study, showed that IL-6 was correlated only with the presence of obesity and ischemic stroke, and only in the group of patients with macrovascular coronary lesions. Obesity is also characterised by a pro-inflammatory status of more reduced intensity, but chronic, termed metabolic inflammation, in which the activated monocytes infiltrate the adipose tissue, and differentiate into resident adipose macrophages which in turn secrete inflammatory cytokines, among which IL-6 [29]. Consequently, obese patients have increased IL-6 values, which results in an increased risk of cardiovascular diseases, insulin resistance, and diabetes mellitus [30]. These data are confirmed by the results of our study, where the IL-6 level in obese patients was indeed correlated with coronary macrovascular disease. Why was it correlated only with this and not with microvascular angina as well? Things are not very clear, but a plausible explanation would be the fact that obesity through its consequences is more frequently associated with the atherosclerotic process, therefore with coronary macrovascular disease. On the other hand, obesity is also associated with OSA (obstructive sleep apnea syndrome) these patients having an increased incidence of cardiovascular disease, especially ischemic coronary heart disease. The pathophysiological mechanisms of OSA involved in ischemic coronary heart disease are represented by chronic activation of the sympathetic system, oxidative stress, endothelial dysfunction, and chronic inflammation. [31,32]. Therefore, the levels of markers, such as CRP, TNF-alpha, IL-6, IL-8, but also that of ET1, are increased, and all being strongly involved in the pathogenesis of atherosclerotic disease. A recently published study, which evaluated the correlation between serum levels of ET-1 and LOX-1(lectin-like oxidized low-density lipoprotein receptor-1), both being involved in the atherosclerotic process, in patients with sleep apnea syndrome and the risk of cardiovascular adverse events, demonstrated a direct relationship between the presence of sleep apnea syndrome and the level of ET-1 and, consequently, a higher cardiovascular risk [32]. Indeed, we did not analyze the prevalence of OSA among the obese patients included in the study, but there is a high probability that they also hide this respiratory pathology, which accentuates inflammation and endothelial dysfunction and implicitly the atherosclerotic process. This may explain the higher value of IL-6 in the group of patients with obstructive coronary heart disease. 

IL-6 is also implicated in the development of ischemic stroke, particularly in the occlusion of small brain vessels. Also, the plasma IL-6 level is a predictor of recurrences as well as of the prognosis of patients with ischemic stroke [33]. It has been proven that about one third of patients with ischemic stroke, but without known coronary disease, have in fact coronary lesions in a proportion of over 50%, and 3% of these are at a risk of developing acute myocardial infarction at one year [34]. In its turn, ischemic coronary disease is associated with an increased risk of stroke, which is one of the most redoubtable extracardiac complications of myocardial infarction [35]. All these statements are valid in the case of ischemic heart disease through macrovascular involvement. The interrelation between microvascular angina and stroke is not currently very well known, but taken independently, studies demonstrate the implication of microvascular cerebral dysfunction as a mechanism in the development of ischemic stroke [36]. The absence of a correlation between the level of inflammation and the occurrence of ischemic stroke among patients with microvascular angina might also be explained by the lower level of inflammation among these patients.

Regarding the influence of inflammatory biomarkers on cardiac functions, on systolic and diastolic function, respectively, we observed no significant correlation between these functions and plasma IL-6 level. This result is again surprising, and contradicts the data obtained in previous studies, which practically supported the hypothesis that, including in patients who are asymptomatic and without associated cardiovascular pathology, IL-6 value is indirectly correlated with the presence of regional left ventricular systolic dysfunction, thus representing a pathogenic link to the development of heart failure [37]. This result could be explained in the first place by the small number of patients included in the study, and in the second place, possibly by the need for a higher IL-6 level. In contrast, ET-1 is correlated with systolic dysfunction expressed by the ejection fraction value only among patients with microvascular angina, not in the second group. The ET-1 value was similar in the two groups, but this correlation might be explained by the more diffuse location of endothelial dysfunction in patients with microvascular involvement, compared to those with atherosclerotic lesions, where the alterations are more localized and are limited to a certain coronary territory, and a correlation with systolic dysfunction in these patients would require a more extensive endothelial dysfunction, therefore a much higher ET-1 level. Thus, it can be stated that the ET-1 level can be a marker of systolic dysfunction in patients with coronary microvascular involvement.

By further analyzing the presence of heart failure, an important complication of ischemic heart disease, it should be emphasized that one of the most important cytokines with effects both regarding the inflammatory and immune response, which is involved in the pathophysiology of heart failure, is IL-6 [38]. It has been proved that the IL-6 level continues to increase progressively in patients with heart failure, with the aggravation of the NYHA functional class, and also, the high level is independently associated with the risk of mortality and hospitalization. In our study, increased IL-6 level was correlated with the NYHA functional class only in patients with microvascular angina, the NYHA IV class being diagnosed only in these patients. 

In contrast, although ET-1 is also a biomarker implicated in the pathophysiological processes of heart failure and its plasma concentrations are generally increased both in heart failure with reduced ejection fraction and in heart failure with preserved ejection fraction and have a negative prognostic role, in our study, ET-1 level was not increased and was not associated with the stage of heart failure. This lack of correlation between ET-1 level and the NYHA functional class is explained precisely by this normal level of ET-1 obtained in our study.

Finally, we want to emphasize that establishing the role of the two biomarkers in the pathophysiology of ischemic heart disease, and of its complications, requires further studies.

## 5. Conclusions

In patients with an increased suspicion of microvascular angina, in whom microvascular dysfunction cannot be invasively assessed, IL-6 levels, unlike the ET-1 levels, in association with other clinical and paraclinical parameters, may be a helpful marker for the diagnosis of microvascular coronary heart disease. 

## Figures and Tables

**Table 1:** Characteristics of the patients included in the study.

Parameters	Details	Global	Group 1 Microvascular Disease (Non-Obstructive Coronary Artery Disease)	Group 2 Macrovascular Disease (Obstructive Coronary Artery Disease)	*p* Value
Number of patients			45 (50%)	45 (50%)	
Age (Mean ± SD)		69.02 ± 9.34	67.37 ± 9.03	70.66 ± 9.46	*p* = ns
Smoking (%)	Yes	30 (33.3)	13 (28.88)	17 (37.77)	*p* = ns
	No	60 (67.6)	32 (71.11)	28 (62.22)	
Symptoms (%-yes)	Typical angina	54 (60)	23 (51.11)	31 (68.88)	*p* = ns
	Atypical pain	19 (21.1%)	13 (28.88)	6 (13.33)	*p* = ns
	Palpitations	37 (41.1%)	25 (55.55)	12 (26.66)	*p* = 0.0101
	Dyspnea	67 (74.4%)	33 (73.33)	34 (75.55)	*p* = ns
	Decreased exercise tolerance	74 (82.2%)	35 (77.77)	39 (86.66)	*p* = ns
No of macrovascular coronary lesions (%)	One-vessel	15 (33.3)	-	15 (33.3)	*p* = ns
	Two-vessel	15 (33.3)	-	15 (33.3)	
	Three-vessel	15 (33.3)	-	15 (33.3)	
Total cholesterol (mg/dL) Mean ± SD		171.9 ±46.55	174.24 ±50.18	169.55 ± 43.05	*p* = ns
LDL cholesterol (mg/dL) Mean ± SD		101.18 ±42.15	105.80 ±43.15	96.57 ±41.09	*p* = ns
HDL cholesterol (mg/dL) Mean ± SD		43.46 ±10.48	43.60 ± 11.85	43.33 ± 9.04	*p* = ns
Triglycerides (mg/dL) Mean ± SD		140.14 ±69.56 (119.5)	125.55 ± 50.88 (117)	154.73 ±82.23 (137)	*p* = ns
Uric acid (mg/dL) Mean ± SD		6.76 ±2.08	6.61 ±1.96	6.91 ± 2.2	*p* = ns
CRP (mg/dL) Mean ± SD		1.13 ± 1.26	1.24 ± 1.67	1.01 ± 0.65	*p* = ns
IL-6 * (pg/mL) Mean ± SD		19.66 ± 55.09 (7.7)	12.36 ± 16.36 (7.5)	26.95± 75.9 (8)	*p* = ns
Log IL-6 Mean ± SD		0.8528 ± 0.5809	0.7760 ± 0.5664	0.9297 ± 0.5913	*p* = ns
ET-1 * (pmol/L) Mean ± SD		1.67 ± 0.5 (1.7)	1.63 ± 0.42 (1.7)	1.7 ± 0.57 (1.7)	*p* = ns
Log ET-1 Mean ± SD		0.20 ± 0.12 ()	0.19 ± 0.11 (0.23)	0.21 ± 0.13 (0.23)	*p* = ns
Diabetes mellitus (%)	Yes	41 (45.6%)	18 (40)	23 (51.11)	*p* = ns
	No	49 (54.4%)	27 (60)	22 (48.88)	
Obesity (%)	Yes	64 (71.1%)	32 (71.11)	32 (71.11)	*p* = ns
	No	26 (28.9%)	13 (28.88)	13 (28.88)	
PPH of ACS (%)	Yes	33 (36.7%)	5 (11.11)	28 (62.22)	*p* < 0.0001
	No	57 (63.3%)	40 (88.88)	17 (37.77)	
	STEMI	15 (45.5%)	2 (4.44)	13(28.88)	*p* < 0.0001
	NSTEMI	14 (42.4%)	2(4.44)	12 (26.66)	
	UA	4 (12.1%)	1 (2.22)	3 (6.66)	
	Without	57 (63.3%)	40 (88.88)	17 (37.77)	
PAD (%)	Yes	14 (15.6%)	5 (11.11)	9 (20)	*p* = ns
	No	76 (84.4%)	40 (88.88)	36 (80)	
CHF/LVF (%)	Yes	68 (75.6%)	28 (62.22)	40(88.88)	*p* = 0.0070
	No	22 (24.4%)	17 (37.77)	5(11.11)	
AFi (%)	Yes	48 (53.3%)	30 (66.66)	18 (40)	*p* = 0.0201
	No	42 (46.7%)	15 (33.33)	27 (60)	
	Permanent	14 (15.6%)	9 (20)	5 (11.11)	*p* = ns
	Persistent	10 (11.1%)	6 (13.33)	4 (8.88)	
	Paroxysmal	24 (26.7%)	15 (33.33)	9 (20)	
	Without	42 (46.7%)	15(33.33)	27(60)	
Ischemic stroke (%)	Yes	26 (28.9%)	11 (24.44)	15 (33.33)	*p* = ns
	No	64 (71.1%)	34 (75.55)	30(66.66)	
CKD (%)	Yes	22 (24.4%)	9 (25)	13 (28.88)	*p* = ns
	No	68 (75.6%)	36 (75)	32 (71.11)	
Anxiety-depressive disorder (%)	Yes	34 (37.8%)	18 (40)	16 (35.55)	*p* = ns
	No	56 (62.2%)	27(60)	29 (64.44)	

IL-6—interleukin 6; ET-1—endothelin 1; ACS—acute coronary syndrome; PPH—personal pathological history; UA—unstable angina; PAD—chronic peripheral artery disease; CHF—congestive heart failure; LVF—left ventricular failure; AFi—atrial fibrillation; CKD—chronic kidney disease; ns—not significant. * The normality conditions were not met.

**Table 2:** Correlation between serum IL-6 and ET-1 levels and the degree of coronary involvement, explained by the number of coronary lesions.

Parameter	Number of Coronary Lesions				*p* Value
	without	One-Vessel	Two-Vessel	Three-Vessel	
IL-6 (pg/mL) Mean ± SD	12.36 ± 16.36 (7.5)	10.06 ± 10.41 (7.7)	12.71 ± 19.17 (6.6)	58.08 ± 126.76 (12.10)	*p* = 0.030
Log IL-6 Mean ± SD	0.7760 ± 0.5664	0.7752 ± 0.4852	0.8335 ±0.4597	1.1803 ± 0.7412	*p* = 0.018
SD-standard deviation, *p* value obtained in the Kruskal–Wallis test and Mann–Whitney tests.					
ET-1 (pmol/L) Mean ± SD	1.63 ± 0.42 (1.7)	1.78 ± 0.7 (1.7)	1.57 ± 0.41 (1.6)	1.76 ± 0.58 (1.7)	*p* = 0.7316
Log ET-1 Mean ± SD	0.19 ± 0.11 (0.23)	0.22 ± 0.14 (0.23)	0.18 ± 0.12 (0.20)	0.22 ± 0.14 (0.23)	*p* = 0.7316

**Table 3:** The relationship between IL-6 and ET-1 values and patients’ age.

	Global	Group 1—Microvascular Disease	Group 2—Macrovascular Disease
IL-6–age	Rho 0.283 *p* = 0.0075	Rho 0.301 *p* = 0.0459	Rho 0.230 *p* = 0.1277
Mean Age		67.37 ± 9.03	70.66 ± 9.46
ET-1–age	Rho 0.0963 *p* = 0.3635	Rho 0.0827 *p* = 0.5835	Rho 0.0944 *p* = 0.5313

**Table 4:** The correlation between the IL-6/ET-1 value and left ventricular function.

	Global	Group 1—Microvascular Disease	Group 2—Macrovascular Disease
IL-6-LVEF%	Rho −0.186 *p* = 0.0789	Rho −0.263 *p* = 0.0813	Rho −0.121 *p* = 0.4225
ET-1-LVEF%	Rho −0.203 *p* = 0.0561	Rho −0.440 *p* = 0.0035	Rho 0.0261 *p* = 0.8625
IL-6–diastolic dysfunction	Rho 0.128 *p* = 0.2255	Rho 0.0637 *p* = 0.6728	Rho 0.197 *p* = 0.1905
ET-1–diastolic dysfunction	Rho 0.0915 *p* = 0.3881	Rho 0.265 *p* = 0.0783	Rho −0.123 *p* = 0.4159

LVEF%—left ventricular ejection fraction.

**Table 5:** Interrelation between IL-6/ET-1 and the development of atrial fibrillation comparatively between the two groups.

	Group 1-Microvascular Disease		Group 2-Macrovascular Disease	
	with AFi	without AFi	with AFi	without AFi
IL-6-ET-1	30 patients- rho 0.193 *p* = 0.2980	15 patients- rho 0.418 *p* = 0.1181	18 patients- rho 0.161 *p* = 0.505	27 patients- rho 0.147 *p* = 0.4542
Spearman correlation coefficient (R), and *p* > 0.05 was considered statistically significant.				

AFi—atrial fibrillation; IL-6—interleukin 6; ET-1—endothelin 1.

**Table 6:** The correlation between the serum levels of IL-6/Log IL-6 and the presence of associated comorbidities in the patients included in the study.

		IL-6				Log IL-6			
		Mean	Standard Deviation	Median		Mean	Standard Deviation	Median	
Diabetes mellitus	yes	14.61	22.94	8.00	*p* = 0.8174	0.8216	0.5667	0.9031	*p* = 0.64
	no	23.88	71.77	7.50		0.8790	0.5970	0.8751	
Obesity	yes	24.51	64.66	8.10	*p* = 0.0353	0.9377	0.5886	0.9085	*p* = 0.0288
	no	7.72	7.90	6.35		0.6439	0.5140	0.8024	
PAD	yes	20.82	34.63	7.55	*p* = 0.6047	0.7749	0.7610	0.8779	*p* = 0.58
	no	19.45	58.26	7.75		0.8672	0.5465	0.8893	
CKD	yes	14.30	22.53	7.35	*p* = 0.3312	0.7832	0.5703	0.8662	*p* = 0.52
	no	21.39	62.14	8.10		0.8754	0.5866	0.9085	
Ischemic stroke	yes	39.69	97.12	9.55	*p* = 0.0497	1.0791	0.6541	0.9793	*p* = 0.0176
	no	11.52	17.68	7.55					

yes—with the mentioned comorbidity; no—without the mentioned comorbidity; PAD—chronic peripheral artery disease; CKD—chronic kidney disease. Due to the fact that IL-6 does not comply with normal conditions, logs were calculated.

**Table 7:** The correlation between the serum levels of ET-1/Log ET-1 and the presence of associated comorbidities in the patients included in the study.

		ET-1				Log ET-1			
		Mean	Standard Deviation	Median		Mean	Standard Deviation	Median	
Diabetes mellitus	yes	1.61	0.29	1.60	*p* = 0.3190	0.20	0.08	0.20	*p* = 0.31
	no	1.72	0.63	1.70		0.20	0.15	0.23	
Obesity	yes	1.71	0.54	1.70	*p* = 0.5658	0.21	0.12	0.23	*p* = 0.56
	no	1.59	0.41	1.67		0.18	0.11	0.22	
PAD	yes	1.85	0.53	1.75	*p* = 0.0994	0.25	0.11	0.24	*p* = 0.09
	no	1.64	0.50	1.60		0.19	0.12	0.20	
CKD	yes	1.76	0.62	1.70	*p* = 0.4442	0.22	0.13	0.23	*p* = 0.44
	no	1.64	0.46	1.67		0.19	0.12	0.22	
Ischemic stroke	yes	1.71	0.55	1.70	*p* = 0.3319	0.21	0.14	0.23	*p* = 0.33
	no	1.66	0.49	1.60		0.20	0.11	0.20	

yes—with the mentioned comorbidity; no—without the mentioned comorbidity; PAD—chronic peripheral artery disease; CKD—chronic kidney disease. Due to the fact that ET-1 does not comply with normal conditions, logs were calculated.
